# Neurophthalmological conditions mimicking glaucomatous optic neuropathy: analysis of the most common causes of misdiagnosis

**DOI:** 10.1186/s12886-016-0395-x

**Published:** 2017-01-10

**Authors:** Diego Torres Dias, Michele Ushida, Roberto Battistella, Syril Dorairaj, Tiago Santos Prata

**Affiliations:** 1 Department of Ophthalmology, Federal University of Sao Paulo, Sao Paulo, Brazil; 2 Glaucoma Unit, Hospital Medicina dos Olhos, Sao Paulo, Brazil; 3 Department of Ophthalmology, University of Sao Paulo, Sao Paulo, Brazil; 4 Department of Ophthalmology, Mayo Clinic, 4500 San Pablo Road, Jacksonville, FL 32224 USA

**Keywords:** Optic disc, Neuro-ophthalmology: diagnosis, Intraocular pressure

## Abstract

**Background:**

To analyze the most common neurophthalmological conditions that may mimic glaucomatous optic neuropathy and to determine which most often lead to misdiagnosis when evaluated by a glaucoma specialist.

**Methods:**

We reviewed the charts of consecutive patients with optic neuropathies caused by neurophthalmological conditions screened in a single Eye Clinic within a period of 24 months. Within these enrolled patients, we selected the eyes whose fundoscopic appearance could resemble glaucoma based in pre-defined criteria (vertical cup-to-disc ratio ≥0.6, asymmetry of the cup-to-disc ratio ≥0.2 between eyes, presence of localized retinal nerve fiber layer and/or neuroretinal rim defects, and disc haemorrhages). Then, color fundus photographs and Humphrey Visual Field tests (HVF) of these eyes were mixed with tests from 21 consecutive glaucomatous patients (42 eyes with normal tension glaucoma). These images were mixed randomly and a masked glaucoma specialist was asked to distinguish if each set of exams was from a patient with glaucoma or with a neurophthalmologic condition.

**Results:**

Among the 101 eyes (68 patients) enrolled with neurophthalmological diseases, 16 (15.8%) were classified as conditions that could mimic glaucoma. The most common diagnoses were ischemic optic neuropathy (25%), compressive optic neuropathy (18.7%) and hereditary optic neuropathy (18.7%). Based on the analysis of fundus photographs and HVF tests, 25% of these were misdiagnosed as glaucoma (two ischemic optic neuropathies and two congenital optic disc anomalies). Conversely, 11.9% of the glaucomatous neuropathies were misdiagnosed as neurophthalmological disorders. Overall, the glaucoma specialist correctly diagnosed 84.5% of the eyes.

**Conclusions:**

Some neurophthalmological disorders can mimic glaucoma. In our study, isquemic and compressive optic neuropathies were the ones that most often did so. Almost one quarter of the eyes were misdiagnosed when evaluated by a glaucoma specialist, which can lead to inadequate management and influence the prognosis of these patients.

## Background

Glaucoma is characterized by retinal ganglion cell degeneration, alterations in optic nerve head topography, and associated visual field (VF) loss. Although elevated intraocular pressure (IOP) remains the most important known risk factor for the development and progression of glaucomatous optic neuropathy, a significant proportion of the cases may present with IOPs in the normal range [1–6].

**Fig. 1:**
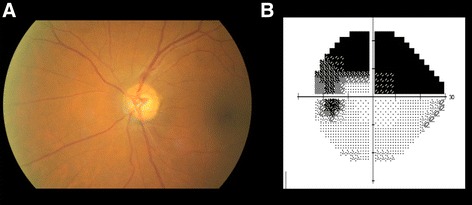
Optic disc findings resembling glaucomatous optic neuropathy in a patient with anterior ischemic optic neuropathy: note violation of the ISNT rule with thinning of the inferior neuroretinal rim, sectorial pallor and arteriolar narrowing (**a**). HVF of the same eye showing superior arcuate defect (**b**)

**Fig. 2:**
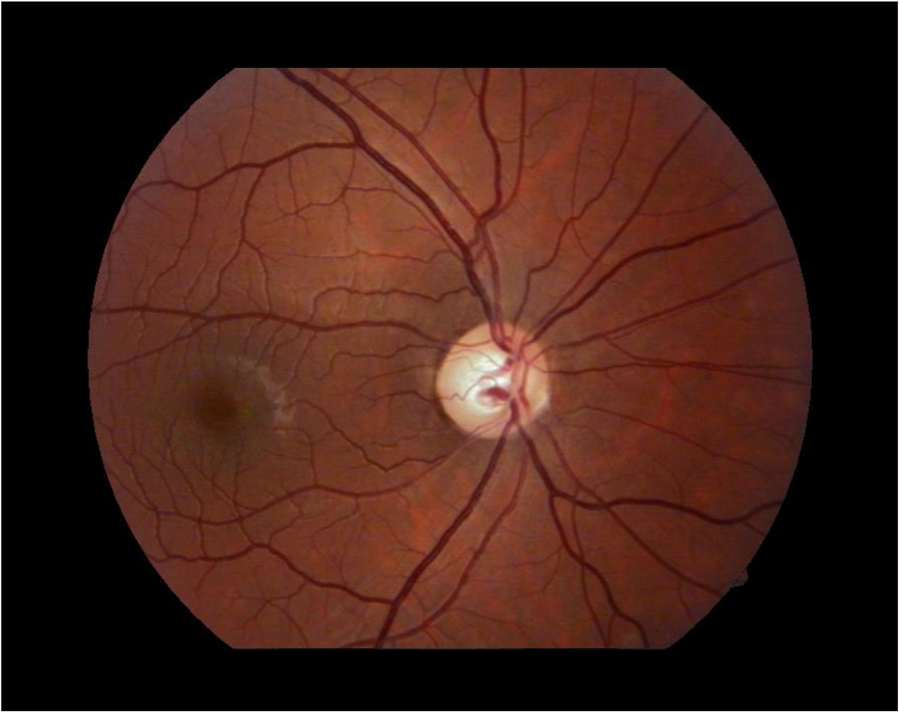
Optic disc cupping with generalized neuroretinal rim pallor in late-stage Leber hereditary optic neuropathy in a young male with central visual field loss and reduced visual acuity

**Fig. 3:**
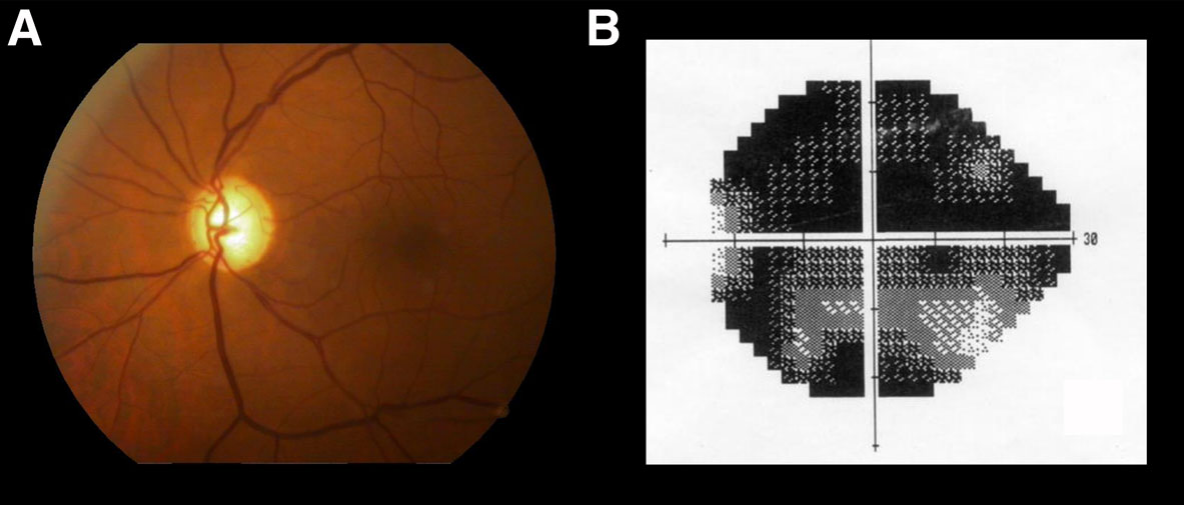
Disc cupping and pallor (**a**) associated with compressive lesion of the intracranial portion of the left optic nerve caused by a dolichoectatic internal carotid artery. Reduced visual acuity, loss of the central visual field (**b**) and neuroretinal rim pallor indicated the need for neuroimaging investigation

Since IOP is within the normal range in eyes with normal-tension glaucoma (NTG), a definitive diagnosis is not always straightforward in these cases, and it’s important to consider all the differential diagnoses. While having in mind other forms of glaucoma, it is necessary to exclude cases of primary open angle glaucoma (POAG) with wide IOP fluctuations, steroid-induced glaucoma, cases of intermittent IOP increase (e.g. uveitis), pigmentary glaucoma (in older people) and others [7]. When thinking about forms of non-glaucomatous neuropathy, one should consider especially those that may present with optic disc cupping (besides visual field loss), such as anterior ischemic optic neuropathies (AION) (Fig. 1), hereditary optic neuropathies (Fig. 2), those associated with compressive lesions (Fig. 3) and demyelinating optic neuritis.

To distinguish glaucomatous and non-glaucomatous disc cupping can be challenging, especially in eyes with IOP within the normal range. In this context, some studies have tried to determine possible discriminating parameters to add clinicians in this task. These studies often focus on clinical data gathered from patients with one specific subgroup of neurophthalmological conditions, like compressive neuropathies, for example [8], or on the need for neurological assessment and neuroimaging in these cases, which remains controversial [9]. Nevertheless, there are scant data when it comes to neuropththalmological diseases in general that resembles glaucoma on daily practice, and especially those that can most often be misdiagnosed as NTG. Since the knowledge of this information could assist handling these challenging cases, we sought to investigate the most common neurophthalmological conditions that may mimic NTG and to determine which most often lead to misdiagnosis when evaluated by a glaucoma specialist.

## Methods

This cross-sectional protocol adhered to the tenets of the Declaration of Helsinki and was approved by the Institutional Review Board. In addition, written informed consent was obtained from all participants.

### Patients

We reviewed the charts of consecutive patients with optic neuropathies caused by neurophthalmological conditions screened in a single Eye Clinic within a period of 24 months. A consecutive set of patients with NTG followed at the same institution was included. The diagnosis of nonglaucomatous optic neuropathy was made by a single experienced neurophthalmologist (RB) based on clinical examination and ancillary exams, such as perimetry, retinography and neuroimaging tests. The diagnosis of NTG was made by a single glaucoma specialist (TSP) based on clinical examination and the presence of signs of glaucomatous optic neuropathy (GON) and characteristic VF loss. All included patients had undergone a comprehensive ophthalmological examination at baseline and performed ancillary exams according to the specialist’s discretion. Exclusion criteria were significant media opacity (precluding proper fundus examination) and the presence of any other ophthalmological condition that could affect the optic nerve or the VF. All included eyes had a maximum untreated IOP <21 mmHg on at least two separate occasions.

Signs of GON were defined as vertical cup-to-disc ratio ≥0.6, asymmetry of the cup-to-disc ratio ≥0.2 between eyes, presence of localized retinal nerve fiber layer and/or neuroretinal rim defects, and disc haemorrhages. Characteristic glaucomatous VF loss was defined as three or more points in clusters with a probability of <5% (excluding those on the edge of the field or directly above and below the blind spot) on the pattern deviation plot, a pattern standard deviation index with a probability of <5%, or a glaucoma hemifield test with results outside the normal limits. Indices for VF test reliability were set at fixation loss <20%, false-negative <33% and false-positive <15%.

The color fundus photographs of all patients with stablished nonglaucomatous optic neuropathy diagnosis were simultaneously evaluated by two glaucoma specialists for identification of those whose fundoscopic appearance resembled glaucoma was based on signs suggestive of GON (described above). In cases of disagreement, the opinion of a third examiner was used for adjudication.

### Data analysis and main outcome measures

After the patients’ diagnoses had been stablished by the specialists and the cases of nonglaucomatous optic neuropathy that resembled glaucoma had been selected by the glaucoma specialists, color fundus photographs (Midriatic retinography; Visucam Lite, Carl Zeiss Meditec; AG07740, Jena, Germany) and standard achromatic perimetry tests results (Humphrey Visual Field Analyzer, Sita Standard, 24-2; Carl Zeiss Meditec; Dublin, CA) of the nonglaucomatous eyes were mixed randomly with those from the NTG patients and reviewed by a different glaucoma specialist (SD) in a masked fashion. The observer was asked to determine whether each subset of images was glaucoma or a nonglaucomatous optic neuropathy case based only on these data (retinography and VF test results). No other demographic or clinical characteristic (such as age, race or IOP) was provided for the examiner. Main outcome measures were: (1) identification of the neurophthalmological conditions that could mimic glaucoma based on the fundus aspect; (2) identification of the conditions that most often led to misdiagnosis (based on the nonglaucomatous optic neuropathy cases that were incorrectly classified as glaucoma); (3) quantification of the ability of the glaucoma specialist to discriminate between glaucomatous and nonglaucomatous eyes (based on the percentage of correct answers).

## Results

We enrolled 101 eyes with nonglaucomatous optic neuropathy from 68 consecutive patients and 42 eyes with NTG from 21 patients. When comparing demographic characteristics between groups, we found NTG patients to be significantly older and with a higher prevalence of Asian descendants (*p* < 0.01). Regarding ocular findings, NTG patients had lower IOP values on average (*p* < 0.01). It is important to emphasize that all glaucomatous patients were under medical treatment. In addition, when comparing VF status between groups, those with neurophthalmological conditions had worse mean deviation values than those with glaucoma (*p* = 0.01). Complete demographic and ocular data are shown on Table 1.

**Table 1:** Comparison of demographic and ocular characteristics between patients with neurophthalmological conditions and normal-tension glaucoma

Parameters^a^	NO Group (*n* = 68)	NTG Group (*n* = 21)	*P* value
Age (years)	44.6 ± 20	56.6 ± 13.8	*p* < 0.01
Gender (%; F/M)	53/47	68/32	*p* = 0.16
Race (%; C/A/Others)	79/2/19	43/35/22	*p* < 0.01
IOP (mmHg) ^b^	17 (14.7, 18.7)	13 (12.7, 14.0)	*p* < 0.01
CCT (μm)	513.5 ± 41.9	529.1 ± 32.9	*p* = 0.14
MD Index (dB)	−6.5 (−10.3, −3.9)	−3.4 (−4.1, −2.3)	*p* = 0.01
PSD Index (dB)	5 (2.2, 8.5)	3.2 (2.2, 7.2)	*p* = 0.09

*NO* neurophthalmological conditions, *NTG* normal-tension glaucoma, *F* female, *M* male, *C* caucasian, *A* asian, *CCT* central corneal thickness, *MD* mean deviation, *PSD* pattern standard deviation

^a^Normally distributed variables represented by mean ± standard deviation; non-normally distributed variables represented by median (first quartile, third quartile)

^b^All normal-tension glaucoma patients were under medical treatment

Among the eyes with neurophthalmological diseases, 16 eyes (15.8%) were considered as conditions that could mimic glaucoma based on the fundus aspect. Looking at the most common diagnoses that filled the GON fundoscopic criteria, we found 4 (25%) cases of non-arteritic ischemic optic neuropathy, 3 (18.7%) cases of tumoral compressive optic neuropathy and 3 (18.7%) cases of hereditary optic neuropathy.

Regarding the neurophthalmological conditions that most often led to misdiagnosis as glaucoma, non-arteritic ischemic optic neuropathies and congenital optic disc anomalies were the most common diagnosis (two cases of each diagnosis). Finally, when considering the diagnostic ability of the glaucoma specialist, we found a diagnostic accuracy of 84.5%. More specifically, 11.9% of the glaucomatous neuropathies were misdiagnosed as neurophthalmological disorders, while 25% of the nonglaucomatous optic neuropathies were misdiagnosed as glaucoma.

## Discussion

Discriminating glaucomatous from nonglaucomatous neuropathy can be a difficult task in clinical practice even for experienced professionals. Although glaucoma is the main cause of disc cupping, 20% of the patients can be misdiagnosed [10]. The results of this study support that nonglaucomatous optic neuropathies can mimic glaucoma specially when IOP is within the normal range, leading to misdiagnosis even when evaluated by a glaucoma specialist based on retinography and perimetry. Additionally, our study provides information about how often nonglaucomatous optic neuropathies may mimic NTG and the conditions that most frequently do so.

There are scant data about conditions that most often lead to misdiagnosis between NTG and nonglaucomatous optic neuropathies. The few previously published studies have pointed out isquemic, compressive and hereditary optic neuropathies as possible causes of nonglaucomatous cupping [7, 8, 10–15]. These studies investigated specific parameters that could distinguish glaucomatous from nonglaucomatous cupping and their potential underlying mechanisms, but focusing solely on one specific optic neuropathy at a time. In this study, we had the opportunity to evaluate a large population of consecutive patients with miscellaneous nonglaucomatous optic neuropathies, highlighting those that most often mimic glaucoma and lead to misdiagnosis when evaluated by a glaucoma specialist. Using fundoscopic criteria, the most common diagnoses were isquemic, compressive and hereditary optic neuropathies, in this order of frequency. Looking at cases that were misdiagnosed as glaucoma, isquemic optic neuropathy and congenital anomalies were the most frequent ones. We believe these results may help in the differential diagnosis of glaucoma cases within normal pressure ranges.

We believe that the differentiation between glaucomatous and non-glaucomatous optic neuropathies is both clinically and economically relevant. In this context, clinicians often have to decide whether or not to request neuroimaging for patients with disc cupping and IOP within the normal range. The incorrect diagnosis of neurophthalmolgical conditions can not only lead to an unnecessary treatment with topical hypotensive drops of a nonprogressive hereditary optic neuropathy, for example, but also to more serious scenarios, such as a late diagnosis of a treatable intracranial tumor. Although this is not the aim of our study, some authors have suggested age <50 years, visual acuity <20/40, visual field defects respecting vertical midline, pallor of neuroretinal rim, asymmetrical loss of color vision and relative afferent pupillary defect as clinical parameters that should be evaluated when considering neurologic evaluation [8, 15], while others suggested considering neuroimaging screening in all patients with NTG [16]. We understand that all clinical parameters discussed so far must be individually considered when making the decision of whether or not to submit the patient to neuroimaging screening. Additionally, based on our findings, one should keep in mind the most frequent differential diagnosis and the most common conditions that lead to misdiagnosis while making this decision.

A secondary purpose of our study was to evaluate the ability of a glaucoma specialist to discriminate glaucomatous from nonglaucomatous neuropathy based on color fundus photographs and VF results. In our study, 88.1% of the glaucoma cases and 75% of the optic neuropathies cases were correctly classified. Other studies reported 75–80% accuracy in diagnosing glaucoma and a lower than 50% accuracy in diagnosing other optic neuropathies [10, 11]. However there are some methodological differences between our study and those previously mentioned that must be pointed out. First, in our study, the masked reader made the diagnosis based not only on the optic disc characteristics, but also on the perimetry data. Furthermore, the cases in our study only needed to be classified as glaucoma or not glaucoma, whereas the other previous studies included other differential diagnoses. We believe these significant differences preclude a straight comparison between the results of these studies and our findings.

It is important to emphasize some specific characteristics and limitations of our study. First, all patients were recruited in one single center and the evaluation of the mixed set of images was performed by one single examiner. Even though we had a large sample of more than 100 consecutive eyes with nonglaucomatous optic neuropathies and the examiner was an experienced glaucoma specialist, these potential sources of bias should be considered while interpreting our results. Second, the masked reader only had access to retinography and perimetry results, which does not correspond to the daily practice, where the ophthalmologist has access to other important clinical information (like age, visual acuity, pupillary reflexes and neurologic symptoms). Although this was not the main focus of our study, this fact should be taken into consideration, as it might have underestimated the diagnostic ability of the glaucoma expert. It is also important to highlight that the option of asking a glaucoma specialist to evaluate the mixed set of images was made because previous studies have already reported that NTG and neurophtalmological conditions are frequently misdiagnosed and this differential diagnosis represents a challenging situation in clinical daily practice. Considering a challenging diagnostic situation, a higher frequence of misdiagnosis would be expected if non-specialists were asked to evaluate those patients. Therefore, we believe that the specialists opinion represents the most accurate evaluation available in clinical practice and would be more appropriate than the general ophthalmologists evaluation in a challenging diagnostic situation.

## Conclusion

In eyes with IOP in the normal range, some neurophthalmological disorders can mimic and be misdiagnosed as glaucoma. Among them, isquemic and compressive optic neuropathies were the most common conditions whose fundoscopic appearance resembled glaucomatous optic neuropathy. Almost one quarter of these eyes were misdiagnosed when evaluated by a glaucoma specialist - isquemic optic neuropathy and congenital anomalies being the causes that most often did so. These can lead to inadequate management and influence the prognosis of these patients.

## Abbreviations

AION: Anterior ischemic optic neuropathies
GON: Glaucomatous optic neuropathy
HVF: Humphrey visual field
IOP: Intraocular pressure
NTG: Normal-tension glaucoma
POAG: Primary open angle glaucoma
VF: Visual field
